# Hypoxia-Induced Down-Regulation of Neprilysin by Histone Modification in Mouse Primary Cortical and Hippocampal Neurons

**DOI:** 10.1371/journal.pone.0019229

**Published:** 2011-04-29

**Authors:** Zheng Wang, Dehua Yang, Xiaojie Zhang, Ting Li, Jia Li, Yu Tang, Weidong Le

**Affiliations:** 1 Institute of Neurology, Ruijin Hospital, Shanghai Jiao Tong University School of Medicine, Shanghai, China; 2 Institute of Health Sciences, Shanghai Institutes for Biological Sciences, Chinese Academy of Sciences and Shanghai Jiao Tong University School of Medicine, Shanghai, China; 3 Department of Neurology, Baylor College of Medicine, Houston, Texas, United States of America; St. Georges University of London, United Kingdom

## Abstract

Amyloid β-peptide (Aβ) accumulation leads to neurodegeneration and Alzheimer's disease (AD). Aβ metabolism is a dynamic process in the Aβ production and clearance that requires neprilysin (NEP) and other enzymes to degrade Aβ. It has been reported that NEP expression is significantly decreased in the brain of AD patients. Previously we have documented hypoxia is a risk factor for Aβ generation in vivo and in vitro through increasing Aβ generation by altering β-cleavage and γ-cleavage of APP and down-regulating NEP, and causing tau hyperphosphorylation. Here, we investigated the molecular mechanisms of hypoxia-induced down-regulation of NEP. We found a significant decrease in NEP expression at the mRNA and protein levels after hypoxic treatment in mouse primary cortical and hippocampal neurons. Chromatin immunoprecipitation (ChIP) assays and relative quantitative PCR (q-PCR) revealed an increase of histone H3-lysine9 demethylation (H3K9me2) and a decrease of H3 acetylation (H3-Ace) in the NEP promoter regions following hypoxia. In addition, we found that hypoxia caused up-regulation of histone methyl transferase (HMT) G9a and histone deacetylases (HDACs) HDAC-1. Decreased expression of NEP during hypoxia can be prevented by application with the epigenetic regulators 5-Aza-2′-deoxycytidine (5-Aza), HDACs inhibitor sodium valproate (VA), and siRNA-mediated knockdown of G9a or HDAC1. DNA methylation PCR data do not support that hypoxia affects the methylation of NEP promoters. This study suggests that hypoxia may down-regulate NEP by increasing H3K9me2 and decreasing H3-Ace modulation.

## Introduction

A neuropathological hallmark of Alzheimer's disease (AD) is the presence of amyloid β-peptide (Aβ) plaques, composed mainly of Aβ1-42, and the excessive accumulation of Aβ1-42 is believed to be responsible for the initial event of neurodegeneration in AD [1]. Previously we have documented that hypoxia is a risk factor for Aβ generation in vivo and in vitro through increasing Aβ generation by altering β- cleavage and γ-cleavage of amyloid precursor protein (APP) and down-regulating NEP, and causing tau hyperphosphorylation [2], [3]. NEP is a synaptic enzyme which is found to play a major role in the clearance of Aβ from the brain [4]. The levels of NEP are declined with aging and hypoxic condition, two of the high risk factors for AD [5], [6]. The decrease in NEP expression has been linked to alteration in Aβ clearance leading to Aβ accumulation, which is considered to be an important event contributing to the development and progression of AD [7].

Hypoxia is one of the major common pathophysiology risk factor for diabetes,stroke, hypertension, atherosclerosis, AD and other diseases [8]. Patients suffering from cerebral ischemia and stroke in which hypoxic conditions occur are much more susceptible to AD [9]. Hypoxia inducible factor (HIF) is one of the early response molecules to hypoxic condition and HIF and its pathways participate in many pathological processes of diseases including AD [2], [10], and over 300 genes are regulated by HIF [11]. Several studies including ours have demonstrated that hypoxia increases the production of Aβ [2], [10], decreases the clearance of Aβ from brain [12] and increases tau phosphorylation [13]. Epigenetic changes play a key role in hypoxia-mediated gene expression change at transcriptional levels [14]. The methylation of CpG islands in the promoter regions of genes can result in the silenced expression [15]. Post-translational modification of histones by regulating cellular gene expression plays a critical role in sustained CpG island methylation [16]. Further, it has been reported that DNA methyltransferase inhibitor 5-Aza reduced the level of H3-K9 methylation in the promoter region of tumor suppressor gene independently of cytosine methylation [17], suggesting a novel function of 5-Aza in histone modification. It has also been demonstrated that post-translational modification of histones can control gene activity. The methylation and acetylation of lysine residues in histones by specific histone methyltransferases (HMTs) [17] and histone deacetylase (HDACs) [18] have been implicated in the alterations of chromatin structure and gene regulation. The methylation of K9 and demethylation of K4 in histone 3 has been associated with condensed chromatin and the silencing of genes [19], and the deacetylation of lysine in histone 3 has been linked to chromatin condensing and gene silencing [18].

In the present study, we have shown that hypoxia causes significant down-regulation of NEP expression, which can be prevented by methylation inhibitor 5-Aza, HDAC inhibitor VA, and siRNA-mediated knockdown of G9a or HDAC1. Our results suggest that chromatin remodeling of crucial AD-related genes by 5-Aza, VA and G9a or HDAC1 inhibitors could provide new therapeutic strategy for AD.

## Results

### Hypoxia inhibits the expression of NEP at the mRNA and protein levels

**Figure 1:**
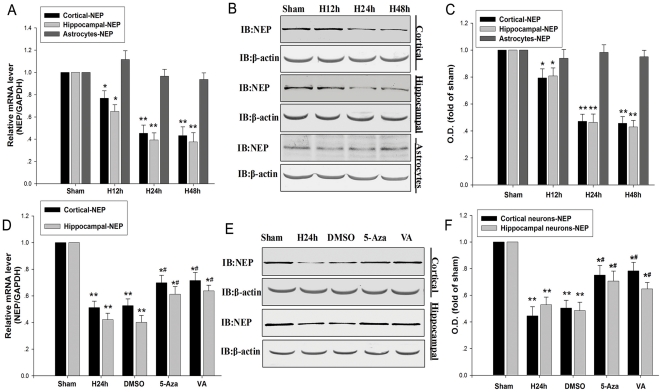
Hypoxia inhibits the expression of NEP at the protein and mRNA levels. (**A**) NEP mRNA expression was determined by real-time PCR. Primary cortical and hippocampal neurons and astrocytes were incubated under the condition of normoxia or hypoxia for 12 h, 24 h, and 48 h. NEP mRNA transcript levels in normoxic control cells were normalized against GAPDH. (**B, C**) Primary cortical and hippocampal neurons and astrocytes were incubated under the condition of normoxia or hypoxia for 12 h, 24 h, and 48 h. The protein of β-actin was blotted as control. (**D**) Primary cortical and hippocampal neurons were incubated under the condition of normoxia or hypoxia for 24 h. Cells were treated with 5-Aza (100 mM) and VA (10 mM) before hypoxia. NEP mRNA transcript levels in normoxic control cells were normalized against GAPDH. (**E, F**) Primary cortical and hippocampal neurons were cultured for 24 h in the presence of 5-Aza, VA or no drug. As control, the protein levels of β-actin were blotted with anti-β-actin antibody. Data are expressed as mean ± SD from four separate experiments. *P<0.05, **P<0.01 as compared with Sham; #*P*<0.05 as compared with H24h. O.D., optical density; Sham, normoxia; H, hypoxia; 5-Aza, 5-Aza-2-deox-ycyti-dine; VA, sodium valproate; GAPDH, glyceraldehydes-3- phosphate dehydrogenase.

In order to detect whether hypoxia can affect the expression of NEP, NEP expression was detected by real-time PCR and Western blot after hypoxic treatment for 12 h, 24 h and 48 h in mouse primary cortical and hippocampal neurons and astrocytes. Our results showed that hypoxia significantly inhibited the expression of NEP both at the mRNA and protein levels (Figure 1A–C) in mouse primary cortical and hippocampal neurons after hypoxic treatment for 24 h and 48 h. But in astrocytes hypoxia did not affect the expression of NEP (Figure 1A–C).

The epigenetic regulators 5-Aza and VA were utilized to examine the epigenetic role in the down-regulation of NEP by hypoxia at the mRNA and protein levels. We found that 5-Aza and VA prevented the down-regulation of NEP by hypoxia (Figure 1D–F). 5-Aza may prevent the down-regulation of NEP via inhibiting cytosine methylation [20], diminishing H3-K9 methylation [17], augmenting H3 acetylation and H3-K4 methylation [21], while VA may prevent the down-regulation of NEP via augmenting H3 acetylation by inhibiting HDACs [22]. These results suggest that either histone/DNA methylation or histone deacetylation is involved in the down-regulation of NEP by hypoxia.

### Histone methylation is involved in hypoxia-induced downregulation of NEP

**Figure 2:**
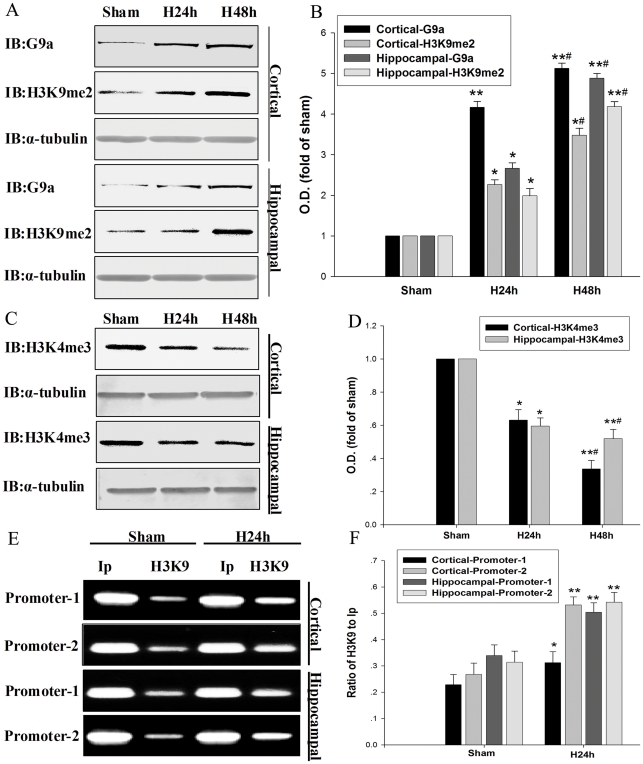
Hypoxia increases histone H3-K9me2 and G9a, but decreases H3K4me3. (A, B) H3K9me2 and G9a of primary cortical and hippocampal neurons were detected by Western blot analysis at 24 h and 48 h after hypoxia. β-actin was used as control. (C, D) H3K4me3 was detected by Western blot analysis at 24 h and 48 h after hypoxia. Data are expressed as mean ± SD from four separate experiments. *P<0.05, **P<0.01 as compared with Sham; #*P*<0.05 as compared with H24 h. (E, F) ChIP and q-PCR analysis were performed with an anti-H3K9me2 antibody and primers amplifying the NEP promoter-1 and promoter-2 regions; the bands illustrate the levels of H3K9me2 in the NEP promoter-1 and promoter-2 regions. Ip represents amplification of total input DNA from whole neurons. H3K9 represents DNA bound to H3K9me2 in the sample. Results from four separate experiments are presented as the ratio of H3K9 to Ip. *P<0.05, **P<0.01 as compared with Sham; Sham, normoxia; H, hypoxia.

Since H3K9me2 is known to cause gene silencing [17], we further determined the H3K9me2 status during hypoxia. We found that H3K9me2 levels were significantly increased in mouse primary cortical and hippocampal neurons after hypoxic treatment for 24 h and 48 h (Figure 2A, B). As H3-K4 trimethylation (H3K4me3) was reported to be involved in gene activation [17], we examined H3K4me3 levels in the mouse primary cortical and hippocampal neurons. As expected, we documented that H3K4me3 was partly diminished following hypoxia in the mouse primary cortical and hippocampal neurons (Figure 2C, D). To determine whether hypoxia-induced H3-K9me2 increase was attributed to NEP down-regulation, we performed the ChIP assay using anti-H3-K9me2 antibody and q-PCR assay. Since NEP expression can be regulated through two distinct promoters called NEP promoter-1 and promoter-2 [23], [24], we measured the interactivity of H3K9me2 with the two promoters. We revealed that under hypoxic condition H3K9me2 levels in the NEP promoter-1 and promoter-2 regions were significantly increased in the cultures of cortical and hippocampal neurons. These results indicate that hypoxia can induce histone methylation in NEP promoters regions, resulting in down-regulation of NEP expression (Figure 2E, F).

**Figure 3:**
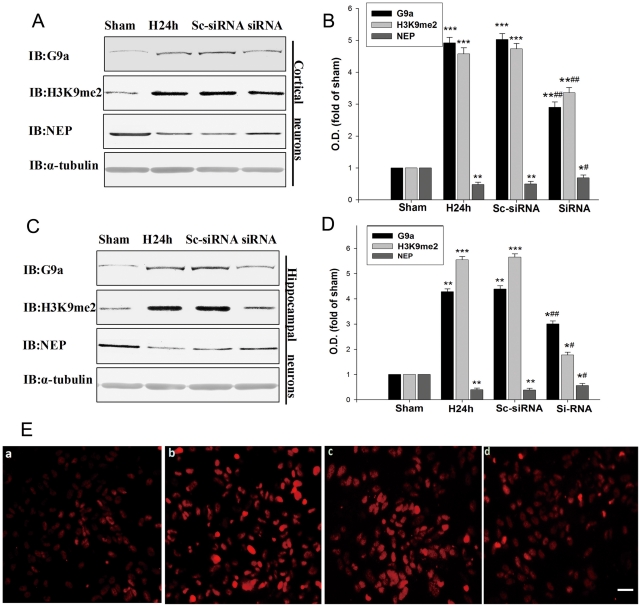
Hypoxia increases histone H3-K9me2 through G9a accumulation. (**A, B, C, D**) Primary cortical and hippocampal neurons were transfected with G9a siRNA, followed by hypoxic exposure for 24 h. G9a, H3K9me2 and NEP protein levels were evaluated by Western blot. The G9a siRNA decreased the expression of G9a, decreased the expression of H3K9me2, and increased the expression of NEP. As control, α-tubulin was blotted. Data are expressed as mean ± SD from four separate experiments. *P<0.05, **P<0.01 and ***P<0.001 as compared with Sham; #*P*<0.05, ##*P*<0.01 as compared with H24 h. (**E**) Immunocytochemical detection of H3K9me2 of primary cortical neurons. (**a**) Sham. (**b**) Hypoxia. (**c**) Transfected with scrambled G9a siRNA as control. (**d**) Transfected with G9a siRNA. Cells were stained with antibody against H3K9me2 followed by a FITC-labeled secondary antibody. The G9a siRNA prevented the hypoxia induced up-regulation of H3K9me2. Magnification: 400 ×. Scale bar, 20 µm. O.D., optical density; Sham, normoxia; H24 h, hypoxia 24 h; Sc-siRNA, scrambled siRNA of G9a; siRNA, siRNA of G9a.

Recently, it has been reported that G9a could modulate H3K9me2 under hypoxic condition [25], [26]. Thus, we investigated whether the H3K9me2 in the NEP promoter-1 and promoter-2 regions were mediated by G9a. Our result showed that the G9a levels were increased in the primary cortical and hippocampal neurons following hypoxic treatment. The change in G9a protein levels was correlated with that of H3K9me2 (Figure 2A, B). To determine whether G9a was crucial for hypoxia-induced NEP down-regulation, we adopted a small interfering (si) RNA strategy. We transfected mouse primary cortical and hippocampal neurons with a siRNA of G9a to knockdown G9a and a scrambled siRNA of G9a as a control. As shown in Figure 3A–D, transfection with G9a siRNAs attenuated the hypoxia-induced increase in G9a expression by 30%–42% and H3K9me2 expression by 34%–63% in the primary cortical and hippocampal neurons, respectively; and G9a siRNAs transfection prevented the hypoxia-induced decrease in NEP by 35% and 24% in the primary cortical and hippocampal neurons, respectively; while transfection with scrambled siRNA of G9a seemed no interference with the expressions of G9a, H3K9me2 and NEP (Figure 3A–D). In addition, we performed an immunocytochemistry study in the neuronal cultures, illustrating that siRNA G9a transfection prevented the hypoxia-induced H3K9me2 up-regulation, which was correlated with the results from Western blot (Figure 3E). Our findings suggest that hypoxia suppresses expression of NEP through the histone methylation by G9a.

### Histone deacetylation is involved in hypoxia-induced down-regulation of NEP

**Figure 4:**
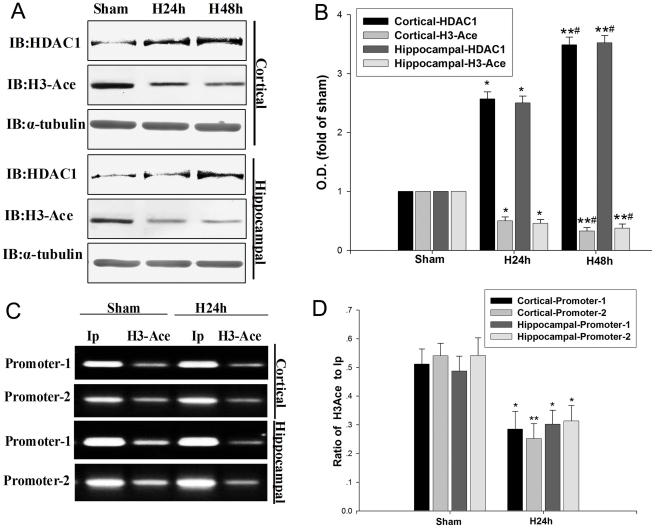
Hypoxia decreases H3-Ace and increases HDAC1 expression. (**A, B**) H3-Ace and HDAC1 of primary cortical and hippocampal neurons were detected at 24 h and 48 h by Western blot analysis. Exposure to hypoxia for 24 h and 48 h decreased H3-Ace, but increased the level of HDAC1. As control, α-tubulin was blotted. Data are expressed as mean ± SD from four separate experiments. *P<0.05, **P<0.01 as compared with Sham; #*P*<0.05, ##*P*<0.01 as compared with H24 h. (**C, D**) ChIP and q-PCR analysis were performed with an anti-H3-Ace antibody and primers amplifying the NEP promoter-1 and promoter-2 regions; the bands illustrate the levels of H3-Ace in the NEP promoter-1 and promoter-2 regions. Ip represents amplification of total input DNA from whole cell lysate. H3-Ace represents DNA bound to H3-Ace in the sample. Results from four separate experiments are presented as the ratio of H3-Ace to Ip. *P<0.05, **P<0.01 as compared with Sham; Sham, normoxia; H24 h, hypoxia 24 h.

Histone deacetylation is known to be involved in gene silencing [17] and HDAC1 is the most important histone deacetylase. Therefore we determined the H3-Ace and HDAC1 status in the primary cortical and hippocampal neurons under the hypoxic condition. We found that histone acetylation was significantly decreased while HDAC1 was significantly increased in the neuronal cultures after treatment for 24 h or 48 h of hypoxia (Figure 4A, B). H3-Ace and HDAC1 expressions were inversely correlated after 24 h or 48 h of hypoxia in the neuronal cultures (Figure 4A, B).

To verify whether histone acetylation under hypoxic condition can result in NEP down-regulation, we performed ChIP assay and q-PCR to determine the interaction of H3-Ace and NEP promoters. As shown in Figure 4C and D, hypoxia decreased the binding of H3-Ace to the NEP promoters. Compared to the input control, promoter H3-Ace was lower in the normoxia-treated cultures than in hypoxia-treated ones. These results indicate that hypoxia could induce histone deacetylation through NEP promoter regions, leading to down-regulation of NEP expression (Figure 4C, D).

**Figure 5:**
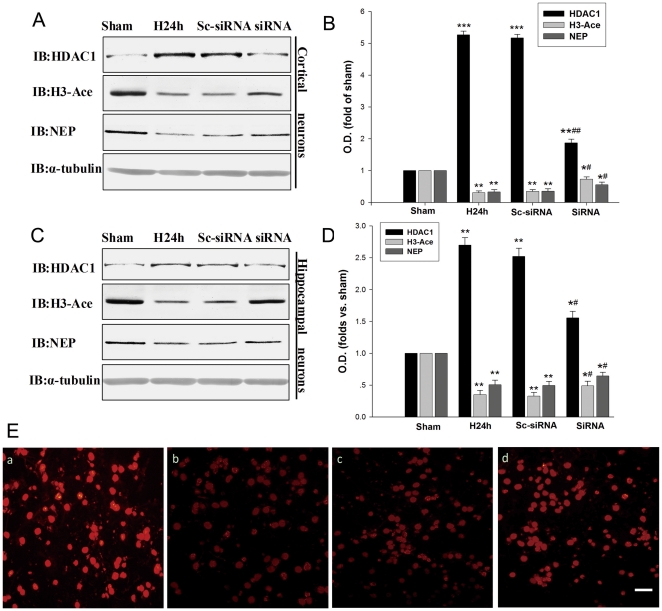
Hypoxia decreases H3-Ace through HDAC1 accumulation. (**A, B, C, D**) Primary cortical and hippocampal neurons were transfected with HDAC1 siRNA, followed by hypoxic exposure for 24 h. HDAC1, H3-Ace and NEP protein levels were evaluated by Western blot. The HDAC1 siRNA decreased the expression of HDAC1, increased the expression of H3-Ace, and increased the expression of NEP. As control, the protein α-tubulin was blotted. Data are expressed as mean ± SD from four separate experiments. *P<0.05, **P<0.01 and ***P<0.001 as compared with Sham; #*P*<0.05, ##*P*<0.01 as compared with H24 h. (**E**) Immunocytochemical detection of H3-Ace of primary cortical neurons. (**a**) Sham. (**b**) Hypoxia. (**c**) Transfected with scrambled HDAC1 siRNA as control. (**d**) Transfected with HDAC1 siRNA. Neurons were stained with antibody against H3-Ace followed by a FITC-labeled secondary antibody. The HDAC1 siRNA prevented the hypoxia induced down-regulation of H3-Ace. Magnification: 400 ×. Scale bars, 20 µm. O.D., optical density; Sham, normoxia; H24 h, hypoxia 24 h; Sc-siRNA, scrambled siRNA of HDAC1; siRNA, siRNA of HDAC1.

It has been reported that HDAC1 can modulate histone deacetylation under hypoxic condition [25], [26]. Thus, we investigated whether HDAC1 contributes to in the histone deacetylation of NEP promoters in association with hypoxia-induced NEP down-regulation. We transfected mouse primary cortical and hippocampal neurons with a siRNA of HDAC1 to knockdown HDAC1 and a scrambled siRNA of HDAC1 as a control. As shown in Figure 5A–D, the treatment with HDAC1 siRNAs significantly attenuated the hypoxia-induced increase in the expression of HDAC1 by 62% and 41% in the primary cortical and hippocampal neurons, respectively. Further, the treatment with HDAC1 siRNAs significantly prevented the hypoxia-induced decrease in the expressions of H3-Ace and NEP by 29%–36% and 27%–31% in mouse primary cortical and hippocampal neurons, respectively (Figure 5A–D). Application of scrambled siRNA of HDAC1 seemed no interference with the expressions of HDAC1, H3-Ace and NEP (Figure 5A–D). In addition, we performed an immunocytochemistry study in the neuronal cultures, illustrating the HDAC1 siRNA prevented the hypoxia-induced H3-Ace down-regulation, which was correlated with the results from Western blot (Figure 5E). These findings suggest that hypoxia can suppress NEP expression through histone deacetylation by HDAC1.

### DNA promoters methylation in hypoxia-induced down regulation of NEP

**Figure 6:**
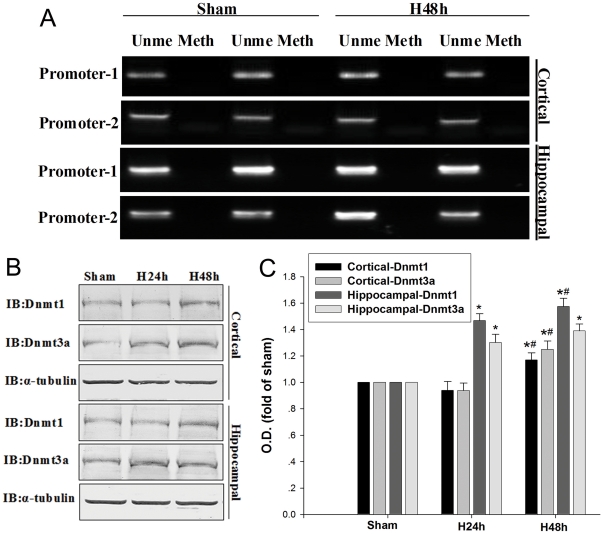
Methylation of NEP promoter regions and DNMT levels. (**A**) Methylation-specific PCR analysis of the NEP promoter-1 and promoter-2 regions were performed with genomi cDNA isolated as described in materials and methods. Methylation was detected by the presence of PCR product amplified by methylation-specific primers in the Meth lane; demethylation was detected using PCR products amplified with unmethylated-specific primers in the Ume lane. (**B, C**) DNMT1 and DNMT3a were detected at 24 h and 48 h by Western blot. As control, α-tubulin was blotted. Data are expressed as mean ± SD from four separate experiments. *P<0.05 as compared with Sham; #*P*<0.05 as compared with H24 h; Sham, normoxia; H48h, hypoxia 48 h; Ume, unmethylated; Meth, methylation.

To determine whether hypoxia can regulate DNA methylation of NEP promoters, we performed methylation-specific PCR assay with the primers designed to amplify NEP promoter regions in the mouse primary cortical and hippocampal neurons. The results showed no obvious change in the levels of unmethylation-specific PCR and methylation-specific PCR under 48 h hypoxic condition as compared with normoxic treatment, suggesting that there was no evidence of hypoxia-induced methylation at CpG sites (Figure 6A). Next we detected the levels of DNMT1 and DNMT3a and found that both of them were significantly increased in the primary hippocampal neurons after hypoxic treatment for 24 h and 48 h, but were only significantly increased in the cortical neurons after hypoxic treatment for 48 h (Figure 6B, C). These results indicate that hypoxia induces significantly changes in DNMT1 and DNMT3a expressions, but the DNA methylation PCR data do not support that hypoxia affects the methylation of NEP promoters.

## Discussion

Abnormal deposition of Aβ in brain is the most significant and important pathological hallmark of AD. Late-onset forms of AD might be primarily attributed to deficiency in the clearance of Aβ rather than its formation [1]. Aβ accumulation has been shown in part by the down regulation of several enzymes associated with degradation, including NEP. The NEP plays a major role in the clearance of Aβ from the brain [4], [27], [27], and over-expression of NEP is found to significantly reduce the Aβ burden in transgenic AD mice [28]. Increasing lines of evidence suggest that NEP levels in the brain declines with aging and chronic hypoxia, and the decreased NEP levels may lead to the reduction in Aβ clearance which may contribute the development and progression of AD [12], [29]. It is well documented that hypoxia is a risk factor for Aβ generation in vivo and in vitro through increasing Aβ generation by altering β-cleavage and γ-cleavage of APP and down-regulating NEP, and possible other pathways [2], [3]. Our results suggest that hypoxia affects the NEP expression both at mRNA and protein levels by regulating the histone methylation and histone deacetylation.

It is increasingly acknowledged that epigenetic phenomena may be a crucial component in the development of complex brain disorders [30]. Indeed, it has been reported that epigenetic mechanisms play an important role in AD [31]. Furthermore, oxidative stress and hypoxia which are the common pathophysiologic contributors in AD [32] have been shown to modulate DNA methylation and histone methylation [33]. Analyses of DNA methylation and histone modulation in genes associated with AD from patients' prefrontal cortex and lymphocytes have revealed abnormal profiles [33]. An age-specific epigenetic drift associated with unusual methylation patterns in late-onset forms of AD has been identified, supporting a potential epigenetic role in the development of AD [34]. Yet, increasing lines of evidence indicate that age and hypoxia may influence epigenetic drift in AD [35].

DNA methylation and demethylation is one of the mechanisms operating in the epigenetic regulation of gene expression. A few studies have shown that DNA methylation of the NEP promoters do not mediate its transcriptional repression in the neuronal lines [36] or in hepatobiliary cells in Alagille syndrome [37]. Meanwhile, hypoxic silencing of RUNX3 may not result from DNA promoter methylation [25]. The epigenetic regulator 5-Aza is known for its ability to inhibit cytosine methylation [20]. Some studies have also demonstrated that 5-Aza is able to cause a regional remodeling of chromatin, by diminishing H3-K9 methylation and augmenting H3 acetylation and H3-K4 methylation, independently of its effects on DNA methylation or gene expression [17], [21]. It has also been reported that hypoxia can result in the down-regulation of MLH1 and RUNX3 by histone H3K9me2 through G9a [25], [26], [38] or through inhibiting histone demethylases JHDM2A or JMJD1A [39]. In our study, we found that hypoxia significantly increases the expressions of DNMT1 and DNMT3a, but does not affect the methylation of NEP promoters (Figure 6A). All the results indicate that 5-Aza may reverse hypoxia-induced down-regulation of NEP by diminishing H3-K9 methylation and augmenting H3 acetylation and H3-K4 methylation.

Post-translational modification of the N-terminal group of histone lysine residues by acetylation or deacetylation can regulate gene activity. HDACs especially HDAC1 play a critical role in sustained histone deacetylation in the gene promoter. Although much attention has focused on the potential function of HDACs in cancer research, recently there have been several studies showing their involvement in neurodegenerative diseases [22]. HDACs inhibitors can enhance the clearance of Aβ from brain [40] by inducing expression of plasmin [12], inhibit the Aβ production and neuritic plaque formation [41], and reverse the contextual memory deficits in a mouse model of AD [42]. In this study, we have demonstrated that hypoxia is able to reduce H3-Ace in the NEP promoters by enhancing HDAC1. However, the role of hypoxia in the neuropathology of AD is yet to be determined.

In summary, we have shown that hypoxia causes significant down-regulation of NEP expression by increasing H3K9me2, H3K4me3 and decreasing H3-Ace modulation, which can be prevented by the epigenetic regulators 5-Aza, HDACs inhibitor VA, siRNA-mediated knockdown of G9a or HDAC1. Chromatin remodeling of crucial AD related genes by 5-Aza, VA, and siRNA-mediated knockdown of HDAC1 or G9a can provide new therapeutic strategy for AD.

## Materials and Methods

### Mouse primary cortical and hippocampal neuron and astrocyte cultures

Animal care and procedures were performed in accordance with the Laboratory Animal Care Guidelines approved by Shanghai Institutes for Biological Sciences of Chinese Academy of Sciences. Permit numbers: SCXK (HU) 2007–0005; SYXK (HU) 2008–0049. This study was approved by Science and Technology Commission of Shanghai Municipality.

The C57 mouse primary cortical and hippocampal neurons and astrocytes were prepared according to previously described procedures. Briefly, primary cortical and hippocampal neurons and astrocytes were grown from dissociated cells derived from the cerebral cortex and hippocampus of 24 h old C57 mouse (Experimental Animal Center of Shanghai). After the cerebral cortex and hippocampus were removed and meninges and microvessels were cleaned, the cortex and hippocampus tissues were cut into 1–2 mm^3^ pieces and then digested with 0.05% trypsin-EDTA (Sigma, USA) and 0.01% DNase I (Sigma, USA) at 37°C for 15 min, then terminated with culture media. The culture medium consisted of Neurobasal™ medium (Gibco, USA), supplemented with 10% FCS (Gibco, USA), 2% (f/c) B-27 supplement (Gibco, USA), 5 mM (f/c) L-glutamine (Sigma, USA), and 10 mg/ml gentamycin (Sigma, USA). After mechanical dissociation, centrifugation, and resuspension, the cells were seeded in 6-well or 24-well tissue culture plates (Coning, USA) pre-coated overnight with poly-L-lysine (Sigma, USA)) at a density of 1 × 10^5^/cm^2^. After culture for 2 weeks, these preparations were referred to as ‘‘neuronal cultures’’ to contain over 90% neurons as illustrated by MAP_2_ immunostaining [43]. Cells were treated with 5-Aza (100 mM) and VA (10 mM) before hypoxia.

For preparation of astrocytes, B-27 wasn't added to the medium. After culture for 2 weeks, the astrocytes in flasks (Coning, USA) were shaken at 190 rpm for 18 h at 37°C to remove microglia and oligodendrocytes, and the adherent cells were allowed to grow for another 3 days. Then, flasks were shaken again under the same conditions to remove the remaining microglia and oligodendrocytes. Next, the attached cells were digested with 0.25% trypsin-EDTA and transferred to 6-well plates at a density of 1.5×10^5^/cm^2^. Using GFAP immunostaining we documented nearly 95% cells GFAP-positive astrocytes [44].

### Hypoxic stress

To study the effect of hypoxia on expression and localization of proteins of interest, the C57 mouse primary cortical and hippocampal neurons and astrocytes were incubated in an O_2_/N_2_/CO_2_ incubator MCO-M (Sanyo, Japan) for 12 h, 24 h or 48 h under 1% O_2_ in accordance with previous reports [45]. The cells were collected 12 h, 24 h or 48 h later, washed twice with 10 ml phosphate buffered Saline (PBS, pH 7.2), scraped into 10 ml of PBS and pelleted at 3,000 g for 5 min. Pellets were resuspended in 6 × vol of 50 mM Tris-1% Triton X-100 (pH 7.4) buffer containing an EDTA free cocktail of protease inhibitors in the concentration suggested by the manufacturer and lysed for 30 min at 4°C. Cell lysates were centrifuged at 12000 g for 20 min and the supernatants were analyzed for protein concentration and used either for Western blotting.

### Real-Time PCR Analysis

Total RNA from cultures was prepared using Trizol reagent (Invitrogen, USA) and digested with RNase-free DNase for 30 min to exclude genomic DNA contamination. For RT-PCR analysis, 2 mg of RNA was transcribed into cDNA with the Reverse Transcription System (Promega, USA) and oligo (dT) primers in a 20-µl volume. The abundance of transcripts in cDNA samples was measured by RT-PCR with primer as follows: NEP forward, 5-’ATCCAACGAATGTGTTAAGGA-3′, reverse, 5-’GTAA GCGTGAGCGCACTAAA-3′; GAPDH forward, 5′-CCATGTTTGTGATGGGTGTGAACCA-3′, reverse, 5′-ACCAGTG GATGCAGGGATGATGTTC-3′ (Sangon Biotech, China). Real-time PCR was performed by Chromo 4 Sequence Detection System (Bio-Rad, USA) utilizing a SYBR Green PCR premix reagent (Toyobo, Japan). The PCR reaction was set up as follows: initial denaturation at 95°C for 10 min, followed by 40 cycles of 15 s at 95°C, 15 s at 58°C and 30 s at 72°C. The levels of NEP expression were normalized to GAPDH.

### Western blot assay

For Western blot assay, cells were washed three times with cold PBS (pH 7.2), and then harvested with a cell scraper, and lysed in cold lysis buffer (modified RIPA) after centrifugation. The protein concentrations were determined by the method of BAC. Equal amounts of protein (60 µg/lane) were separated on polyacrylamide gels and then electrotransferred onto a nitrocellulose membrane (Amersham, UK). After blocking for 3 h in Tris-buffered saline with 0.1% Tween-20 (TBST) and 3% bovine serum albumin (BSA), membranes were incubated overnight at 4°C with primary antibodies: H3K9me2 and H3K4me3 at 1∶500 (Upstate, USA, a generous gift from Prof. Degui Chen, The Shanghai Institute of Biochemistry and Cell Biology, Chinese Academy of Sciences), H3-Ace at 1∶500 (Santa Cruz Biotechnology, USA), NEP at 1∶500 (Santa Cruz Biotechnology, USA), Dnmt1 and Dnmt3a at 1∶500 (Santa Cruz Biotechnology, USA). Membranes were then washed and incubated with alkaline phosphatase conjugated secondary antibodies in TBST for 2 h and developed using NBT/BCIP substrate (Promega, USA). The densities of the bands on the membrane were scanned and analyzed with an image analyzer (Lab Works Software, USA).

### Immunofluorescent staining

For immunofluorescent staining, cells were washed three times in D-Hanks' and then fixed for 15 min at 37°C in 4% PFA. After incubation with PBS containing 0.2% Triton X-100, 5% BSA for 30 min at room temperature (RT), the neurons were incubated with primary antibodies (H3K9me2 at 1∶300, Upstate, USA; H3-Ace at 1∶300, Santa Cruz Biotechnology) overnight at 4°C. After washing, the cells were then incubated with secondary antibody (Cy2-conjugated anti-mouse IgG at 1∶200, Jackson, USA) at RT for 2 h.

### Chromatin immunoprecipitation assay and qPCR Analysis

A ChIP assay was performed using a ChIP assay kit (Upstate, USA) according to the manufacturer's protocol with slight modifications. Briefly, cells were cross-linked with 1% formaldehyde (Sigma, USA) in a culture medium at RT for 10 min. Cells were then suspended in SDS lysis buffer (1% SDS, 10 mM EDTA, 50 mM Tris pH 8.1 and 1 × protease inhibitor) and sonicated on ice. Next, the chromatin solution was precleared, incubated with anti-H3-Ace or anti-H3K9me2 antibodies (Upstate, USA), and immune complexes were eluted. DNA recovered from the immunoprecipitated complex was subjected to PCR with 38 cycles [36]. The primers for the NEP gene promoters were promoter-1: Forward, 5′- TTCCCTGAAGTCAGGAGGTG-3′, reverse 5′-CCTCCCTCCTTCGTTTTCTT-3′ (177bp); promoter-2 forward, 5′-AGATGTGCAAGTGGCGGAAG-3′, reverse, 5′-CGCACCCACAG AGACTCAC-3′ (150 bp); DNA bands were visualized and semi-quantified using the Image J program (NIH, Bethesda, MD, USA).

### Methylation-specific PCR and unmethylation-specific PCR

Genomic DNA was extracted by a standard proteinase K digestion and phenol chloroform procedure. Genomic DNA was modified by EZ DNA Methylation Direct™ Kit (Zymo Research, USA) according to the manufacturer's protocol. After modification, PCR amplifications were performed with 40 cycles at 95°C for 30 s, 57–62°C for 30 s and 72°C for 30 s in a Pelrier Thermal Cycler (Bio-RAD, USA). The primer sequences for the unmethylated products were promoter-1: forward, 5′-TTCCCTGAAGTCAGGAGGTG-3′, reverse, 5′-CCTCCCTCCTTCGTTTTCTT-3′ (177 bp); promoter-2: forward, 5′-AGATGTG CAAGTGGCGGAAG-3′, reverse, 5′-CGCACCCACAGAGACTCAC-3′ (150 bp); The primer sequences for the methylated products were MS-promoter-1: forward, 5′-GAAC TCCGAACGAATAAACG-3′, reverse, 5′-ATTTAGGGAATTGTTTTCGC-3′ (183 bp); MSpromoter-2: forward, 5′-TTCTGGTTCTGTTCTGTTGTGTG-3′, reverse, 5′-TCCCAA CCAATAAACACACCAA-3′ (165 bp).

### Transient transfection and siRNA silencing

One double-stranded siRNAs designed to target G9a and HDAC1, and a scrambled siRNA were synthesized as control (Gene Pharma Co., Ltd, China). Transfection with Lipofectamine 2000 (Invitrogen, USA) was performed according to the manufacturer's protocol. Mouse primary cortical and hippocampal neurons were transfected with 100 nM duplexes targeted to G9a and HDAC1, and a scrambled siRNA were treated as control before hypoxia. G9a and HDAC1 protein expression were evaluated by Western blot after 24 h post-transfection. SiRNA for G9a were designed to target 5′-AAGCTCTAACTGAACAACTAA-3′ and 5′-CACCATGAACATCGATCGCAA-3′, and siRNAs for HDAC1 were designed to target 5′-CACC CGGAGGAAAGTCTGTTA-3′ and 5′-GACGAGTCCTATGAGGCCATT-3′ [25].

### Statistical analysis

Significant differences between experimental groups were determined using one-way analysis of variance (ANOVA) with Sigma Stat. *P* value of less than 0.05 was considered to have significant difference. The quantitative data were obtained from 4 to 5 independent assays with duplication in each assay.
